# Comparison of thiopentone with lidocaine spray vs propofol for laryngeal mask airway insertion at tikur anbessa specialized hospital. A prospective cohort study

**DOI:** 10.1016/j.amsu.2021.102436

**Published:** 2021-06-02

**Authors:** Engidawork Belete, Misrak W/Yahones, Zemedu Aweke, Getahun Dendir, Simeneh Mola, Derartu Neme, Getnet Melaku, Siraj Ahmed, Teshome Regasa, Brook Tesfaye

**Affiliations:** a Diredawa University, College of Medicine and Health Science, Department of Anesthesia, Diredawa, Ethiopia; b Addis Ababa University, College of Medicine and Health Science, Department of Anesthesia, Addis Ababa, Ethiopia; c Dilla University, College of Medicine and Health Science, Department of Anesthesia, Dilla, Ethiopia; d WolaitaSodo University, College of Medicine and Health Science, Department of Anesthesia, Sodo, Ethiopia; e Dilla University, College of Medicine and Health Science, Department of Midwifery, Dilla, Ethiopia

**Keywords:** Topical lignocaine, Propofol, Thiopentone, LMA, **ASA**, American Society of Anesthesiology, **DBP**, Diastolic Blood Pressure, **ECG**, Electro Cardio Graph, **ETB**, Ethiopian Birr, **HR**, Heart Rate, **IV**, Intra Venous, **LMA**, Laryngeal Mask Airway, **MAP**, Mean Arterial Blood Pressure, **NIBP**, Non Invasive Blood Pressure, **SBP**, Systolic Blood Pressure, **SPSS**, Statistical Package for Social Sciences

## Abstract

**Background:**

Insertion of laryngeal mask airway (LMA) requires an adequate depth of anesthesia. Optimal insertion conditions and hemodynamic stability during LMA insertion are mainly influenced by the choice of the intravenous induction agent. Propofol was recommended as a standard induction agent for LMA insertion. Due to unavailability and cost for treatment Propofol is not easily availed, thus this study aimed at assessing the effect of thiopentone with lidocaine spray compared to Propofol on hemodynamic change and LMA insertion on the patient undergoing elective surgery.

**Methods:**

Eighty-four participants were followed in a prospective cohort study based on the induction type of either thiopentone-lidocaine group (TL) or Propofol (P). Hemodynamic variables, LMA insertion condition, apneic time, and cost of treatment during the perioperative time were recorded. Data were checked for normality using the Shapiro-Wilk test. Numeric data were analyzed unpaired student's t-test or Manny Whitney test. Categorical data were analyzed by the chi-square test. A p-value ≤ 0.05 was considered a statistically significant difference.

**Result:**

The comparison of data showed that a significant reduction in mean arterial blood pressure (MAP) in the Propofol group during the first 10 min. The MAP at first minute after LMA insertion was 78.4 ± 5.5 in the Propofol group compared to 81.8 ± 5.6 in thiopentone-lidocaine group p < 0.001. the mean MAP at 5th and 10th minutes after LMA insertion is also significantly lower in the Propofol group compared to the thiopentone-lidocaine group, p < 0.05. There were no statistically significant differences regarding the heart rate change and insertion conditions between the two groups. Mean apneic time was 138 ± 45.8 s in the Propofol group and 85 ± 13.8 s in thiopentone-lidocaine group p < 0.001. Thiopentone-lidocaine group had a lower treatment cost compared to the Propofol group.

**Conclusion:**

Thiopentone with 10% topical Lignocaine is an alternative for the insertion of LMA to Propofol, with better hemodynamic stability and cost-effectiveness.

## Highlights

• Insertion of laryngeal mask airway requires an adequate depth of anesthesia.
• The choice of induction agent influence the optimal insertion conditions of LMA.
• The hemodynamic stability can also be affected by types of induction agent.
• Thiopentone with 10% topical Lignocaine is an alternative for the insertion of LMA.

## 1 Background

The use of laryngoscopy and tracheal intubation were common during the maintenance of the airway which was key importance during any anesthetic procedure. During endotracheal intubation hemodynamics changes, such as tachycardia, hypertension, and arrhythmias can cause myocardial ischemia. Laryngeal mask airway is preferred over endotracheal intubation to prevent adverse cardiovascular effect caused because of laryngoscopy and intubation [1,2]. The cardiovascular response to insertion for LMA is much lower than that of endotracheal intubation, and the incidence of postoperative sore throat is lower after LMA use as compared to endotracheal intubation [3].

Successful insertion of LMA with less undesired effects requires an adequate depth of anesthesia and suppression of the upper airway reflexes [4]. When LMA insertion was tried under light anesthesia coughing, gagging and laryngospasm may result. It also increase the incidence of regurgitation and aspiration [5].

A standard method of providing anesthesia for LMA insertion is with the use of IV Propofol, which has the advantage of inducing anesthesia rapidly and depressing upper airway reflexes. However, Propofol is expensive and painful on injection and associated with a greater degree of ventilator depression and longer apnea than thiopental also causes greater cardiovascular depression than thiopental during induction of anesthesia [6]. Compared to Propofol, thiopentone has the advantage of painless injection and also less incidence of hypotension. Regardless of this, thiopentone does not provide good jaw relaxation which is a vital for LMA insertion. When used alone, it also cause undesirable effect such as laryngospasm, coughing and gagging [7]. The admixture of topical lignocaine were proven to facilitate easy and safe LAM insertion in adults. Topical lignocaine decreased the Propofol dose required for induction. Lower incidence of apnea and fewer hemodynamic changes were observed [8].

Thiopentone alone used for LMA insertion has showed less satisfactory condition than propofol and thiopentone with lignocaine spray [9]. Lidocaine spray and intravenous administration were among various adjuvant drug used together with thiopentone for insertion of LMA to decrease adverse response [[10], [11], [12]].

Due to unavailability and cost for treatment Propofol is not easily availed, thus this study aimed at assessing the effect of thiopentone with lidocaine spray compared to Propofol on hemodynamic change and LMA insertion on the patient undergoing elective surgery.

## 2 Methods

### 2.1 Study design and patients

A Prospective cohort study was conducted at “TikurAnbessa Specialized Hospital"to compare hemodynamic response and LMA insertion condition between the Propofol group (P) and thiopentone-lidocaine (TL) group during Janury20/2017 to April 20/2017. Ethical clearance was obtained from Addis Ababa university ethical review board. Informed consent was taken from patients aged above or 18 whereas, assent was taken from parents or caretakers of a child below 18. The work was reported in line with STROCSS criteria www.strocssguideline.com. The study was registered on research registry with a unique identification number of **researchregistry6780**. All patients aged above 10 years, ASA I and II were included. Patients with any risk of difficult intubation, pregnancy, induction agent used other than study drugs for LMA, a patient who was premedicated with opioids or non-opioid analgesics rather than induction purpose, allergy to study drugs, inhalational agents other than halothane, and anemia was excluded from this study.

### 2.2 Sample size determination and sampling procedure

**Fig. 1:**
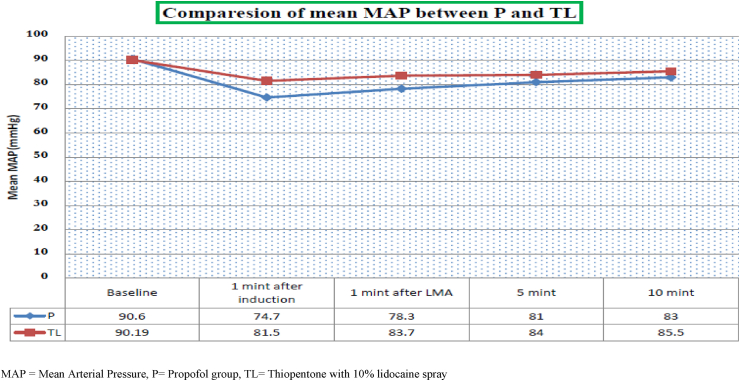
MAP = Mean Arterial Pressure, P= Propofol group, TL = Thiopentone with 10% lidocaine spray.

The sample size was calculated using two independent sample formulas. Based on the previous study, the mean (±SD) apnea time was μ1 = 75.4 ± 7 s in the Propofol group and μ2 = 83 ± 15 s in the thiopentone-lidocaine group [13]**.** Using a 95% confidence interval, power of 80%, and an alpha value of 0.05 the sample size per group is 36. Adding 15% non-response rate and considering n1 = n2, the total sample size is 84 (42 each). From a situational analysis performed before the start of the study, 192 patients aged above 10 years undergo general anesthesia using LMA insertion. Systematic sampling techniques were used to select the required sample size with a sampling interval, k determined by dividing the total population to the required sample size. Using the elective surgery schedule as a sampling frame, participants were recruited for every interval after selecting a random start by lottery methods. Follow up and analysis of study participants were shown in Fig. 1.

### 2.3 Data collection

After getting ethical clearance and permission data were collected by trained personal. Data collectors collected baseline MAP and HR from monitoring used for patient care including a pulse oximeter, ECG, and NIBP. Demographic data such as age, sex, ASA class, and weight were also recorded. Patients were then observed and followed for Hemodynamic during induction time, apnea time, and LMA insertion condition at 1st, 5th, and 10th min after the insertion of the LMA based on their exposure status. Additionally, the cost of treatment was also computed based on data from the pharmacy department cost estimation for drugs. Patient response to LMA insertion was scored based on the scoring system which has six variables **(**i.e. gagging, coughing, jaw relaxation (mouth opening), patient movement, number of attempts to LMA insertion, and laryngeal spasm**)** and 3 point scale [14]**.** After computing for total insertion scorea score of 18 is labeled as excellent, 16–17 as satisfactory, a score 7-16 as poor, and unacceptable for a score of 6.

### 2.4 Data processing and analysis

Data were checked manually for completeness and coded before entered into the Epi Info version 7 and transferred to SPSS-20 to analyze. Data were tested for normality of distribution using the Shapiro Wilk normality test and homogeneity of variance was assessed by Levene's test for equality of variances. Continuous variables were analyzed using an independent *t*-test or Manny Whitney test. A Chi-square test was applied for categorical variables. A p ≤ 0.05 was considered statically significant.

### 2.5 Operational definition

**Apnea time**: The time in seconds from the start of breath-holding to the start of spontaneous breathing.

**Adequate muscle relaxation for insertion of LMA**: loss of motor response to jaw thrust**.**

**Effective LMA insertion:** when there was a response to LMA insertion scoring greater than 16 (excellent or satisfactory), apnea time less than 1 min, and no significant hemodynamic change [15].

**Overall insertion conditions** were assessed according to the modified Scheme of Lund and Stovener

Excellent: No gagging or coughing, no patient movement, or laryngospasm.

Good: Mild to moderate gagging, coughing, or patient movement with no laryngospasm. Poor: Moderate to severe gagging, coughing, or patient movement with no laryngospasm. Unacceptable: Severe gagging, coughing, or patient movement or laryngospasm.

## 3 Result

A total of 84 patients aged above 10 years were enrolled in the study based on their exposure to either Propofol or thiopentone with lidocaine spray for induction of anesthesia for LMA insertion. There was no statistically significant difference between groups on age, ASA class, weight, or sex, p > 0.05 as shown in Table 1.

**Table 1:** Socio-demographic and ASA classes of patients who underwent elective surgery under general anesthesia with LMA.

Variables	TL (n = 42)	P (n = 42)
Gender; M/F	32/10	31/11
ASA class: ASAI/ASA II	37/5	36/6
Age in years (mean ± SD)	18.3 ± 6.6	18.1 ± 6.5
Weight in kg (mean ± SD)	43.3 ± 12.7	42.8 ± 12.8

TL- Thiopentone-lidocaine 10% spray, P= Propofol, SD= Standard deviation.

### 3.1 Hemodynamic changes between groups

**Fig. 2:**
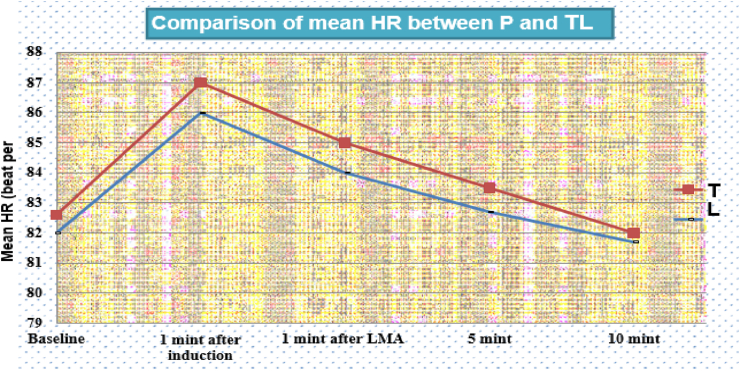
HR = Heart Rate, P= Propofol group, TL = Thiopentone with 10% lidocaine spray.

The baseline mean of MAP of two groups are comparable and statistically not significant. But, following the induction of anesthesia and LMA insertion the mean MAP reduction was higher in the Propofol group compared to the thiopentone-lidocaine group at 1st, 5th, and 10th minutes with p < 0.05. Fig. 2.

**Fig. 3:**
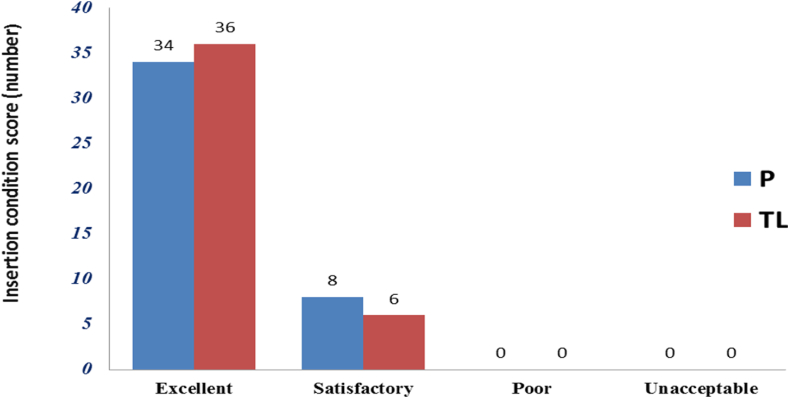
Total distribution of patients' overall insertion condition based on the insertion score between groups.

Similarly, there was no statistically significant difference between group regarding the mean heart rate between the group at 1st, 5th, and 10th minutes after insertion of LMA between groups as shown in Fig. 3 below.

### 3.2 Mean dose of drug consumption andCost

The requirement of the total mean dose of Propofol for induction was 2.8 mg/kg and fentanyl 1.2 mcg/kg to LMA and thiopentone with 10% lignocaine spray 4.6 mg/kg, fentanyl 1.1 mcg/kg and 10% topical lignocaine spray 40 mg the total cost required per case used was ere shown in Table 2 below.

**Table 2:** Comparison of the costs inquired by using thiopentone with 10% topical lignocaine and Propofol drugs to LMA insertion.

Prices in Birr (Ethiopia Birr)		
Drug Type	Cost per case (P)	Cost per case (TL)
Mean weight (kg)	42.8	43.3
Fentanyl (100 mcg) (40 Birr)	20.50	19
Propofol (200 mg) (60 Birr)	32.10	
Thiopentone (500 mg) (15 birrs)		6
Lignocaine (100 gm.) (350 birrs)		0.14
Total cost (per case)	52.6	25.14

P= Propofol group, TL = Thiopentone with 10% lidocaine spray.

### 3.3 Apnea time and patient responses to laryngeal maskinsertion

The mean apnea time in the thiopentone-lidocaine group was 85 ± 13.8 s which was lower than Propofol 138 ± 45.8 s. The difference was statistically significant with a p-value <0.001. Regarding the responses of the patients to LMA insertion, there were no statically significant differences between the two groups on gagging, coughing, mouth opening, laryngospasm, and limb movements. No LMA insertion requires more than two attempts in both study groups as shown in Table 3.

**Table 3:** Adverse responses of the patients to LMA insertion in the thiopentone-lidocaine and Propofol group.

Description	TL group		P group			P_ value
	Grades		Percent	Number	Percent	
		Number				
**Coughing**	Nil	39	92.9	39	92.1	–
	Mild	3	7.1	3	7.9	
**Mouth opening**	Full	40	95.2	39	92.9	.747
	Partial	2	4.8	3	7.1	
**Laryngospasm**	Nil	42	100	42	100	–
**Limb movements**	Nil	38	90.5	36	85.7	0.360
	Mild	4	9.5	6	14.3	
**Gagging**	Nil	38	90.5	37	88.1	0.616
**# ofattempts**	Mild	4	9.5	5	11.9	0.36
	One	37	88.1	37	88.1	
	Two	5	11.9	5	11.9	

P= Propofol group, TL = Thiopentone with 10% lidocaine spray.

### 3.4 Comparison of insertion condition based on insertion scores

Insertion conditions were excellent in 80.9% of the thiopentone-lidocaine group which was lower than Propofol group 85.7%, but the difference is not statistically significant p > 0.05. Fig. 3.

## 4 Discussion

Our study showed that the addition of 10% topical lignocaine spray with thiopentone had a comparable effect with Propofol on Heart rate (HR) change during and after LMA insertion. The propofol group showed a reduction in mean arterial pressure (MAP) from baseline compared to the thiopentone-lignocaine group. There was no difference between groups regarding the insertion condition. The cost of treatment is lower for the thiopentone-lidocaine group compared to the Propofol group.

Similar to this, a study by Patrick Scanlon et al. on topical lignocaine and thiopentone for the insertion of LMA comparison with Propofol for LMA they found that there was no significant difference in heart rate between the two groups, but the decrease in systolic and diastolic blood pressure was significantly greater in the Propofol group (p < 0.05). These might be due to the almost similar dose we used in our study [16].

In contrary to this, the randomized trial by Sengupta Jet al fond mean HR was significantly higher in the thiopentone group immediately after insertion of LMA, and at 1, 3, and 5 min after LMA insertion compared to the Propofol group. The difference might be due to we use topical lignocaine 10% spray with thiopentone [17].

Regarding the change in MAP from baseline our study revealed a decline in MAP from baseline in the Propofol group during and 1st, 5th, and 10th minutes after LMA insertion. Similarly, a decrease in MAP following induction of anesthesia with Propofol is observed during the time of LMA insertion in a study by Townsend R et al. This similarity might be because of the equivalent dose of study drugs in the study. The cardiac depressant effect of Propofol can be attributed to the effect in addition to the effect of Propofol on systemic vascular resistance [18].

Our study reveals thiopentone lidocaine group had a lower apneic time 85 ± 13.8 s compared to 138 ± 45.8 s in the Propofol group with a significant p-value < 0.05. The results were in line with the study by Patrick Scanlon et al. where patients receiving thiopentone and lidocaine had a mean apneic time of 96.1 s and those receiving Propofol had a mean apneic time of 184.9 s [16]. Another study by Mohammad Sadiq et al. also shows the duration of apnea was longer in Propofol group 108sec as compared to thiopentone with lidocaine 10% group 74 s and the difference between the two groups was statistically significant. This similarity might be the relative dose of Propofol used was similar between groups.

Regarding the insertion condition, our result showed no significant difference between groups. There was no significant difference between the two groups concerning the insertion condition of the patients. The insertion condition was excellent in 80.9% in the thiopentone lidocaine group compared to 85.7% in the Propofol group (p > 0.05) [19]. Patrick Scanlon et al. on other hand showed Propofol was superior over thiopentone alone as induction for LMA insertion. Comparing an adverse response to LMA insertion thiopentone had a 76% adverse response to insertion compared to only 26% in the Propofol group. No patient was judged to be inadequately relaxed in the Propofol group and this was less than 11%in the thiopentone group. Similar results showed by Brown GW et al. with a higher incidence of coughing and gagging in the thiopentone group [16,20]. This difference might be related to the use of 40 mg of 10% lidocaine spray before thiopentone in our patients was associated with reduced side effects thiopentone by airway reflex suppression**.**

Besides, the cost for treatment was also lower in the thiopentone lidocaine group compared to the Propofol group. Our study showed that the cost per induction dose for Propofol is 52.6 Ethiopian birr which is expensive than thiopentone lidocaine group 25.14 Ethiopian birr. There were no similar studies to compare cost of treatment.

In conclusion, thiopentone with lidocaine spray is an alternative for induction during LMA insertion. It provides low cost alternative induction techniques for LMA insertion compared to Propofol.

### 4.1 Limitation of the study

Lack of adequate literatures to compare for variable like cost of treatment were the limitation of the study.

## Availability of data and material

Data used in the current study was collected by trained data collectors and authors are willing to share the data upon request. To request the data, contact the first author, EngidaworkBelete,engda20xy@yahoo.com.

## Funding

This work was funded by 10.13039/501100007941Addis Ababa University. The university has no role in the design of the study, collection, analysis, and interpretation of the data and in writing the manuscript.

## Ethical approval

Ethical approval was given from Addis Ababa University college of medicine and health science ethical review board.

## Sources of funding

Addis Ababa University has funded the research project.

The sponsor has no any role other than enhancing staff research and academic activity.

The sponsor didn't take part in any action of the research project other than funding.

## Author contribution

Engidawork Belete, Misrak W/Yahones, Zemedu Aweke, Getahun Dendir, Simeneh Mola, Derartu Neme, and Getnet Melaku contribute to study conception, design, data collection, and performed statistical analysis. Siraj Ahmed, Teshome Regasa, and Brook Tesfaye contributed for interpretation of the result, writing up and prepared manuscript. All the authors read the manuscript and approved the final submission.

## Registration of research studies

1. Name of the registry: Research Registry
2. Unique Identifying number or registration ID: researchregistry6780
3. Hyperlink to your specific registration (must be publicly accessible and will be checked): https://www.researchregistry.com/browse-the-registry#home/

## Guarantor

EngidaworkBelete: engda20xy@yahoo.com Zemedu Aweke: zemeduawoke@yahoo.com.

## Consent

Verbal and written informed consent obtained from each respondent before actual data collection. Issues of confidentiality were maintained by removing any identifiers from the questionnaire. The participant were informed their right to participate and leave the study at any point in time.

## Declaration of competing interest

There is no conflict of interest to declare.
